# BRS1 mediates plant redox regulation and cold responses

**DOI:** 10.1186/s12870-021-03045-y

**Published:** 2021-06-11

**Authors:** Dongzhi Zhang, Yuqian Zhao, Junzhe Wang, Peng Zhao, Shengbao Xu

**Affiliations:** State Key Laboratory of Crop Stress Biology for Arid Areas, College of Agronomy, Northwest A&F University, Yangling, 712100 Shaanxi China

**Keywords:** Serine carboxypeptidase, Cold stress response, Redox, Arabidopsis

## Abstract

**Background:**

Brassinosteroid-insensitive 1 suppressor 1 (BRS1) is a serine carboxypeptidase that mediates brassinosteroid signaling and participates in multiple developmental processes in Arabidopsis. However, little is known about the precise role of BRS1 in this context.

**Results:**

In this study, we analyzed transcriptional and proteomic profiles of Arabidopsis seedlings overexpressing *BRS1* and found that this gene was involved in both cold stress responses and redox regulation. Further proteomic evidence showed that BRS1 regulated cell redox by indirectly interacting with cytosolic NADP + -dependent isocitrate dehydrogenase (cICDH). One novel alternative splice form of *BRS1* was identified in over-expression mutants *brs1-1D,* which may confer a new role in plant development and stress responses.

**Conclusions:**

This study highlights the role of BRS1 in plant redox regulation and stress responses, which extends our understanding of extracellular serine carboxypeptidases.

**Supplementary Information:**

The online version contains supplementary material available at 10.1186/s12870-021-03045-y.

## Background

Serine carboxypeptidases (SCP) are a class of eukaryotic proteolytic enzymes, belonging to the *α/β* hydrolase family. These enzymes contain a highly conserved catalytic amino acid triad, Ser-Asp-His [1]. In Arabidopsis, 54 SCP-like genes have been identified and categorized into three classes [2, 3]. However, the functions of majority of these genes have not been described.

BRS1 belongs to class II of the Arabidopsis SCP family [1]. Its over-expression can suppress the phenotype of brassinosteroid (BR) receptor mutant, *bri1-5*, indicating BRS1 play an important role in BR signaling [4, 5]. In Arabidopsis, *BRS1* has five close homologs, of which three can suppress *bri1-5* developmental defects [6]. In contrast, no significant phenotypes have been identified in single or double mutants of either *BRS1* or its homologs [5–7], indicating *BRS1* and its homologs are functionally redundant in Arabidopsis.

Increasing evidence has revealed that SCP and SCP-like (SCPL) proteins play crucial roles in the regulation of stress responses, including regulating wound healing [8], programmed cell death and response to pathogen infections [9, 10]. BRS1 contains a signal peptide and localizes to the extracellular space [4, 5]. Since the extracellular space is central in regulating plant stress responses [11–13], it is likely BRS1 might mediate some of these signaling pathways. Then this hypothesis keeps in largely unknown in current understanding.

In this study, transcriptomic and proteomic analyses demonstrate a role of BRS1 in cold stress responses. Our results indicate BRS1 participates in redox regulation via interaction with cICDH, shedding new light on the function of SCPs in plant stress responses.

## Results

### Seedling developmental phenotypes of BRS1

**Fig. 1:**
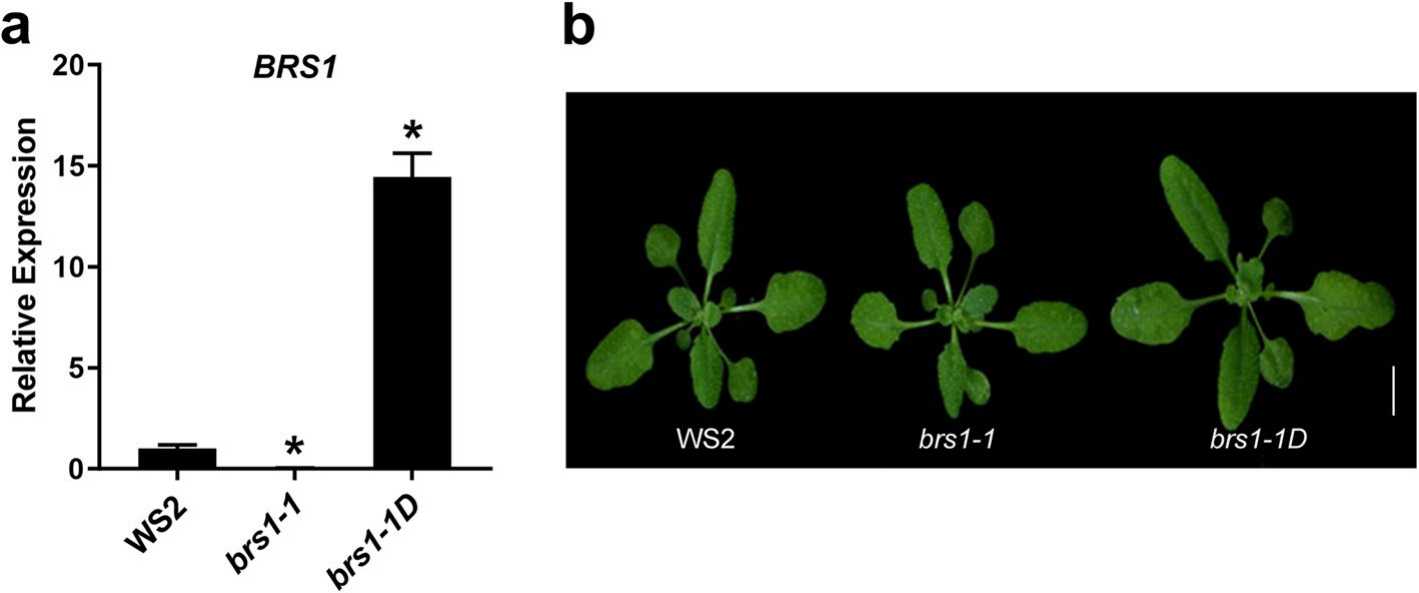
The seedling phenotypes in wild type and *BRS1* mutants. **a** The relative expression level of *BRS1* in wild type WS2, *brs1-1* and *brs1-1D.* All results are shown as mean ± standard deviation (SD) from three biological replicates with qRT-PCR. The asterisks indicate a statistically significant difference (Student’s *t*-test, *p < 0.05). **b** The seedling phenotypes of WS2, *brs1-1* and *brs1-1D* at 14 days after germination (DAG). Scale bar = 1 cm

The mutant *brs1-1D* has an enhancer in *BRS1* promoter [4, 5], resulting in about 15 times increase in *BRS1* transcription compared to that in wild type WS2 (Fig. 1a), and showing larger rosette leaves (Fig. 1b) and a longer hypocotyl (Supplementary figure S1). In contrast, BRS1 knockout mutant, *brs1-1*, showing a significant transcriptional decrease in *BRS1* (Fig. 1a), but no significant phenotype was observed [4–7].

### Analysis of the transcriptomes of BRS1 seedlings

**Fig. 2:**
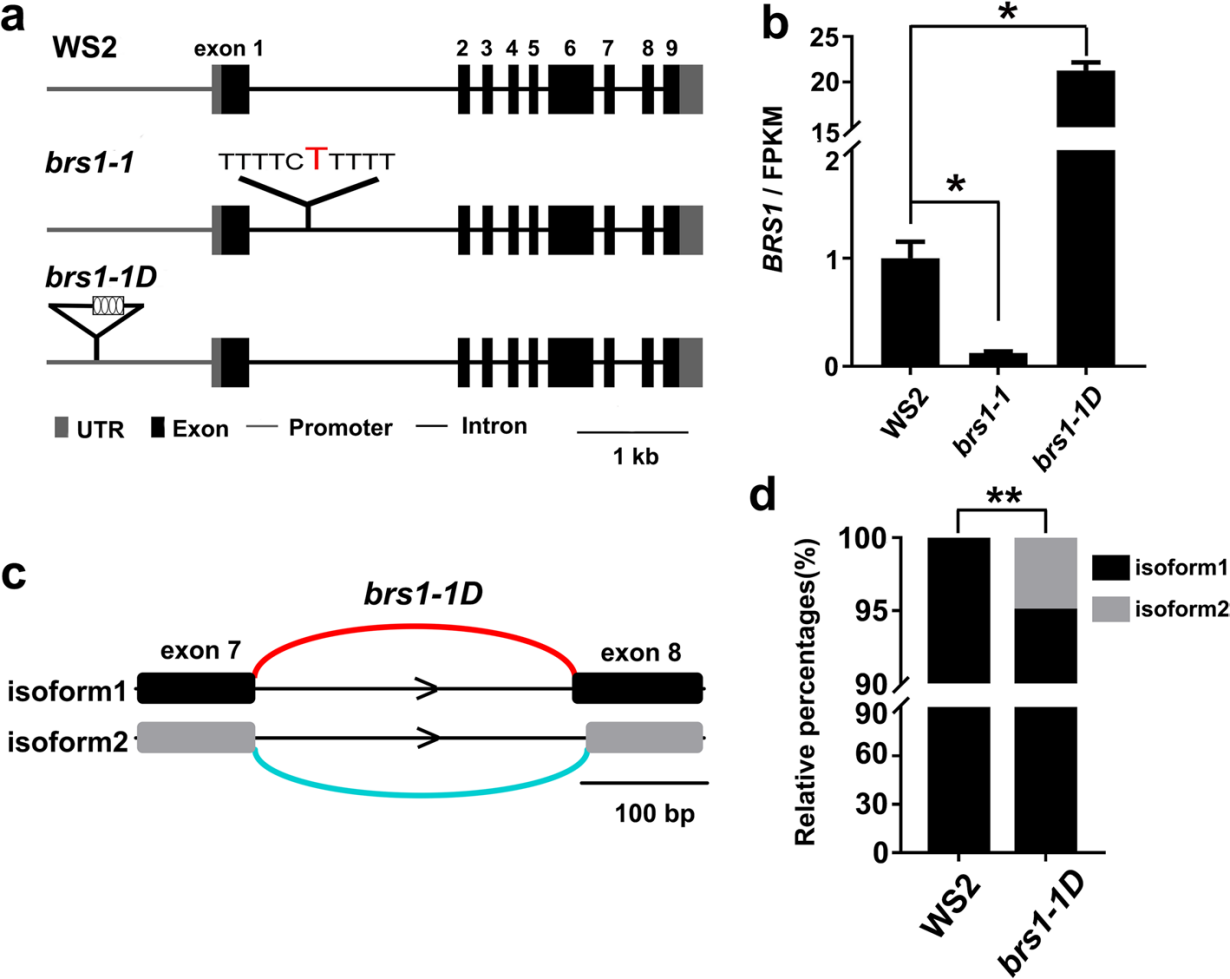
Differences in gene sequences and *BRS1* transcription in wild type and *BRS1* mutants. **a** The sequence difference of *BRS1* in WS2, *brs1-1* and *brs1-1D*. The number above the exon represent the exon 1–9 from 5’ to 3’. A single thymidine base (red) is inserted in the first intron of *brs1-1*, and the 4 × 35S enhancer (four ellipses in black box) is inserted in the promoter of *BRS1* in *brs1-1D*. **b** Transcription level (FPKM value) of *BRS1* in WS2, *brs1-1* and *brs1-1D*. All data were from RNA-seq and shown as mean ± SD. Three biological replicates were quantified. The asterisks indicate a statistically significant difference (Student’s *t*-test, *p < 0.05). **c** Alternative splicing event identification in *brs1-1D*. “isoform1” is the form in the TAIR 10 annotation. “isoform2” is newly identified in our result. **d** The relative percentages of isoform1 and isoform 2 in WS2 and *brs1-1D*. The asterisks indicate a statistically significant difference (**FDR-adjusted *pvalue* < 0.01)

The RNA sequencing was performed on WS2, *brs1-1* and *brs1-1D* seedlings, and showing the *BRS1* in *brs1-1* has an insertion of one thymidine at position 533 in the first intron (Fig. 2a) and a significant decrease in transcription (Fig. 2b). Additionally, no increased transcription was observed on the homologs of *BRS1* in *brs1-1* (Supplementary figure S2 ), which indicates there is no transcriptional feedback loop among *BRS1* and its homologs. In contrast, the transcriptional level of *BRS1* increased by around 20 times in the *brs1-1D* mutant (Fig. 2b), which is consistent with the presence of four copies of CaMV 35S enhancers inserted in the promoter region of *BRS1* [5].

Notably, one novel splice variant isoform was detected in *brs1-1D*, which had an alternative 5′ splice site (Fig. 2c). Compared with the isoform annotated in the TAIR 10 database, novel isoform had 11 bp less on the 5′ site of exon 8. This new splice product made up 5.02% of the total *BRS1* transcripts in *brs1-1D* (Fig. 2d), but was not detected in wild type and *brs1-1* mutant, indicating new transcriptional product exist in *brs1-1D* besides its transcriptional level change in *brs1-1D*.

### BRS1 mediates multiple plant stress responses

With RNA sequencing, a total of 21,873 transcripts were identified, of which 180 were assigned as differentially expressed genes (DEGs, fold change ≥ 2.0, FDR‐adjusted *p*‐value < 0.05, FPKM ≥ 1) in *brs1-1D* compared to wild type, including 114 increased genes and 66 decreased genes (Table S1). In contrast, there were no DEGs identified in *brs1-1* seedlings, except *BRS1* itself (Table S1).

**Fig. 3:**
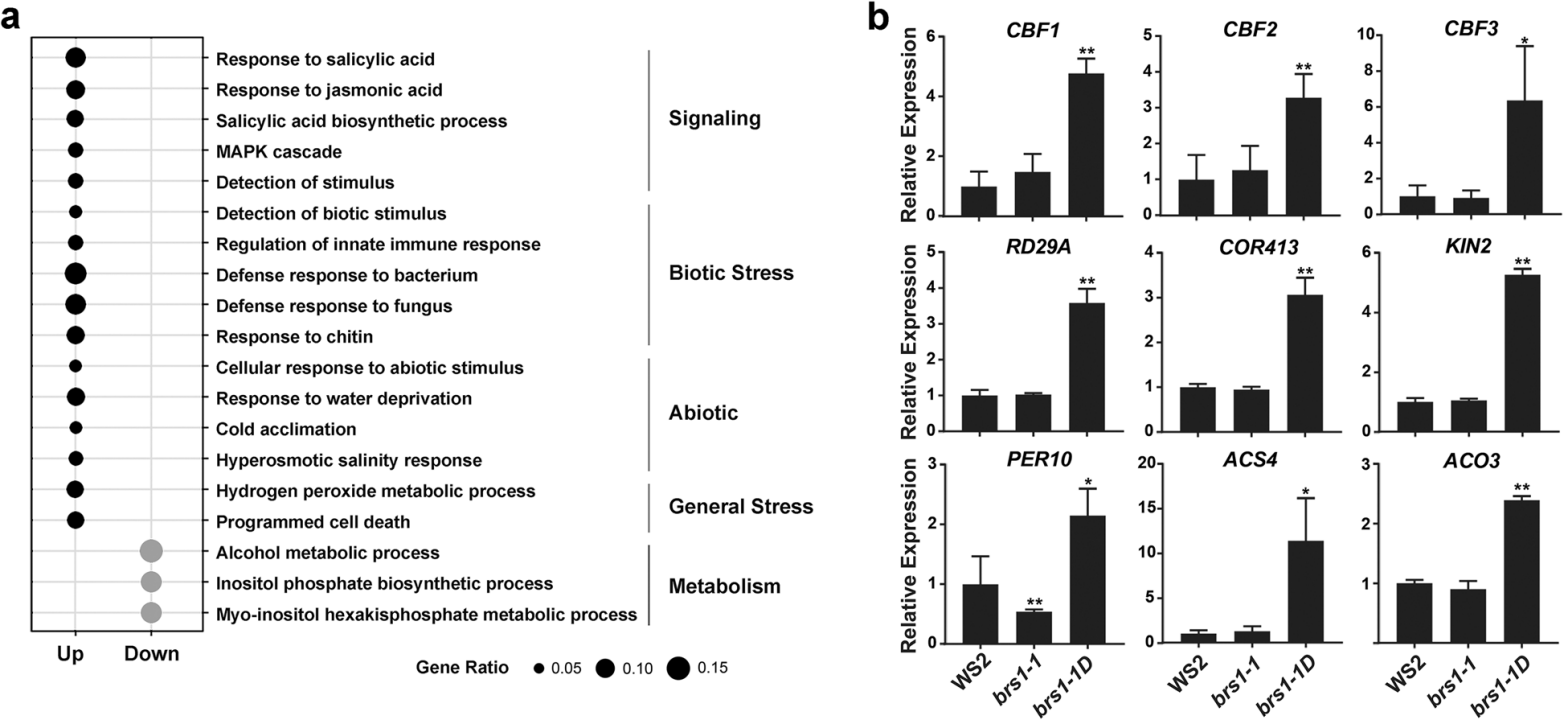
The genes involved in abiotic response. **a** Gene ontology enrichment analysis of DEGs between *brs1-1D* and WS2 (*q*-value < 0.01). The black and grey dots represent GOs that are up-regulated and down-regulated in *brs1-1D* respectively. The terms enriched GOs are divided into five categories. Gene ratio indicates the number of DEGs from different term, divided by the total number of DEGs. **b** The qRT-PCR detection of cold related genes (*CBF1*, *CBF2*, *CBF3*, *RD29A*, *COR413*, *KIN2*), redox related gene (*PER10*) and ethylene synthesis related genes (*ACS4*, *ACO3*). These genes were significant up-regulated in *brs1-1D* compared to WS2, confirmed the results from RNA sequencing (Table S1). All results are presented as mean ± SD from three biological replicates at least. The asterisks indicate a statistically significant difference (Student’s *t*-test, **p < 0.01, *p < 0.05)

Gene Ontology (GO) enrichment analysis of DEGs from *brs1-1D* revealed significant enrichment of terms associated with responding to salicylic acid and jasmonic acid (Fig. 3a). We also found marked enrichment for genes involved in both biotic (innate immune responses, bacterium, fungus and chitin) and abiotic (water deprivation, cold and hyperosmotic salinity) stresses (Fig. 3a). In agreement with these findings, genes associated with redox regulation and cell death were also enriched. These results strongly suggest that BRS1 participates in environmental stress responses.

To support the role of BRS1 in stress response, qRT-PCR was performed on the genes involved in redox regulation, ethylene synthesis and cold response (Fig. 3b). This analysis verifies the transcriptional changes in different genotypes (Table S1), supporting that BRS1 has a role in stress response.

### Cold tolerance is significantly enhanced in *brs1-1D*

**Fig. 4:**
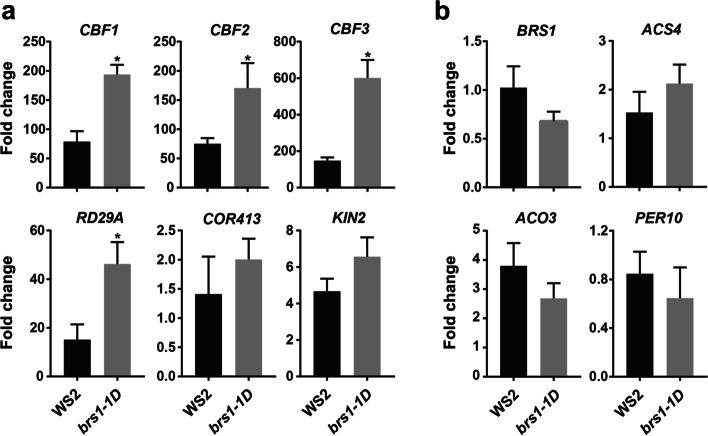
The *brs1-1D* shows a higher cold sensitivity. The qRT-PCR was performed in each genotype with and without freeze treatment (4 ℃ for 3 h). The columns represent the fold change of gene expression after freeze treatment compared to untreated control. All results are shown as mean ± SD from three biological replicates at least. The asterisks indicate a statistically significant difference (Student’s *t* -test, *p < 0.05). **a** Cold signaling genes responses with freeze treatment. **b** Stress related genes response with freeze treatment. Including ethylene synthesis related genes (*ACS4* and *ACO3*) and redox related gene *PER10*

Notably, three core genes involved in cold signaling, the C-repeat binding factors *CBF1*, *CBF2* and *CBF3* [14], all displayed significantly increased transcription in *brs1-1D* (Fig. 3b). We therefore checked the response of these genes to cold stress. The result showed all these cold transcriptional factors increased two times transcriptional level in *brs1-1D* (Fig. 4a) after a freeze treatment (4 °C for 3 h), compared to those in wild type plant. Consistently, cold signaling downstream genes *RD29A* [15], *COR413* [16], *KIN2* [17] showed similar increase trend, while redox and ethylene signaling genes kept the same transcriptional level with wild type after freeze treatment (Fig. 4b), indicating the BRS1 has a role in enhancing the cold sensitivity.

**Fig. 5:**
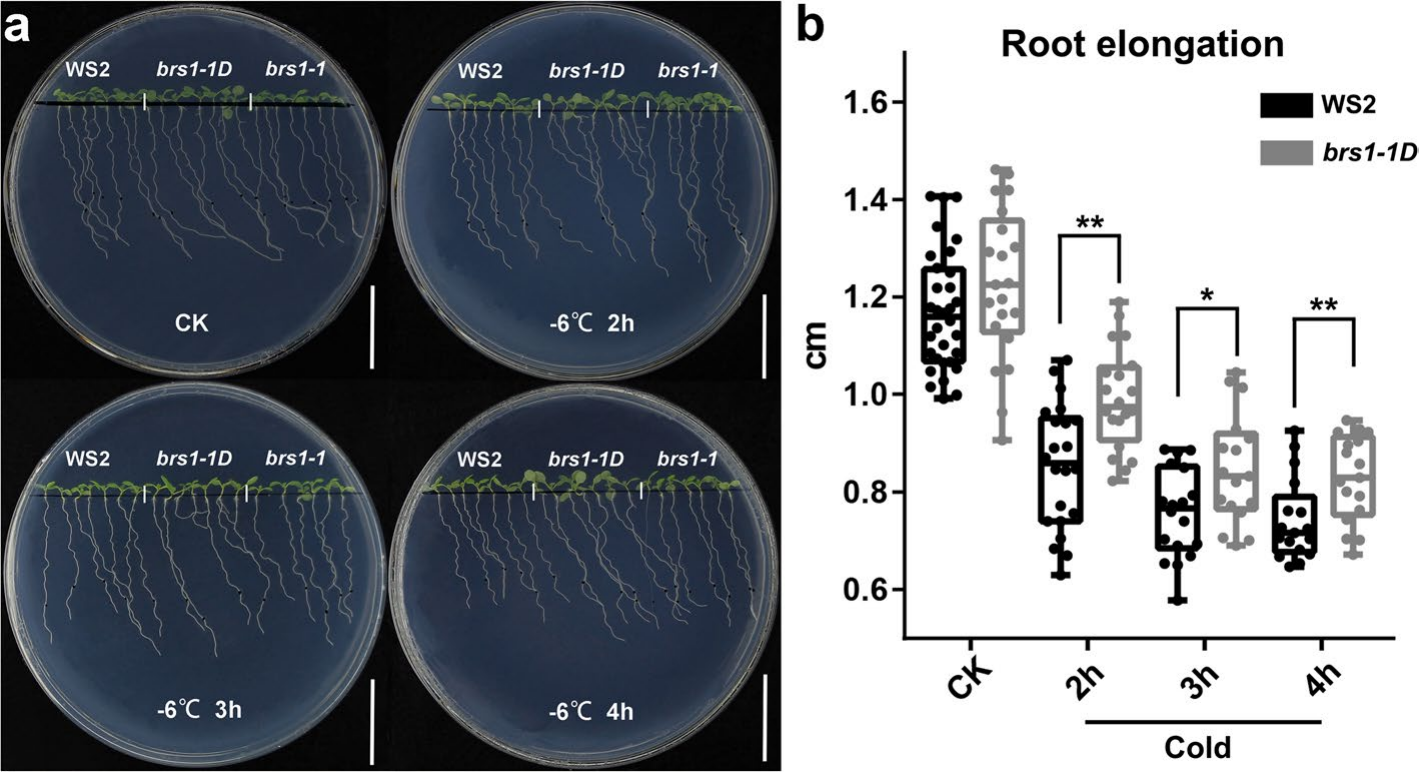
The phenotypes of seedling in cold treatment. 7 DAG seedlings were shifted to -6 °C for 2, 3 and 4 h and then returned to normal conditions (22 °C) for 36 h. **a** The phenotypes of WS2, *brs1-1* and *brs1-1D* grown on 1/2 MS medium under normal conditions and with cold treatment for 2 h, 3 h and 4 h. Scale bar = 2 cm. The black dots on the plate represent the growth position of the seedlings before the cold treatment. **b** The root elongation in WS2 and *brs1-1D* after a severe cold treatment. The root elongation was measured at 36 h after cold shock. Data is from three biological replicates. The same results were repeated at least two times. The asterisks indicate a statistically significant difference (Student’s *t*-test, ** p < 0.01)

Further, the phenotypes of seedling after severe cold shock (-6 °C for 2 h, 3 h and 4 h) were investigated (Fig. 5a), we found the root elongation of *brs1-1D* was significantly longer than the wild type at 36 h after cold shock (Fig. 5b), suggesting that the over-expression of *BRS1* contributes to a higher cold tolerance.

### BRS1 regulates redox-related proteins

**Fig. 6:**
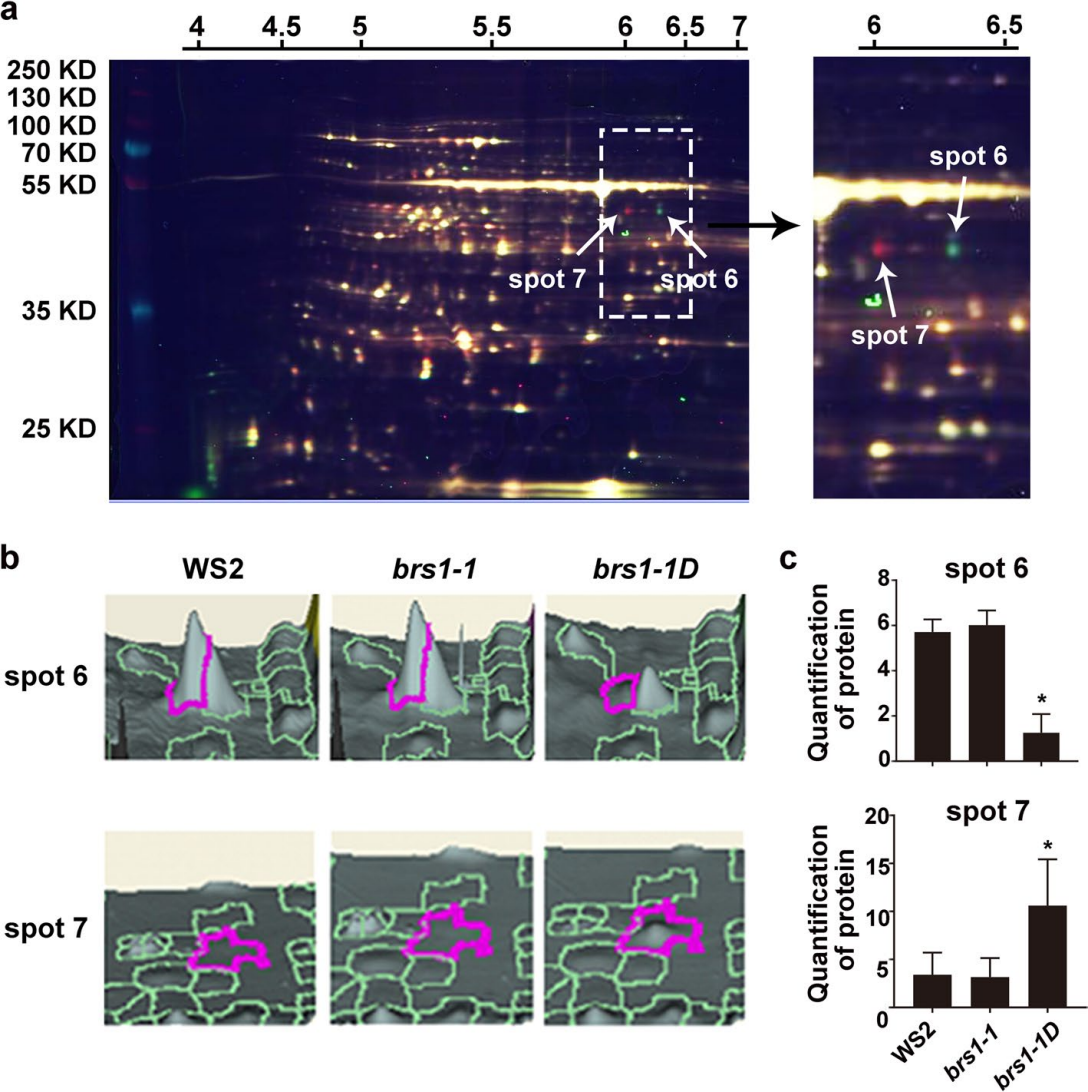
Identification of two isoforms of cICDH and comparison of protein expression levels. **a** Representative 2D-DIGE image. Samples from WS2, *brs1-1* and *brs1-1D* were labeled with Cy2 (blue), Cy3 (green), and Cy5 (red), respectively. The x- and y-axis represent the isoelectric point and molecular weight of the protein, respectively. White arrows indicate differential protein spots. **b** Expression levels of two differential protein spots in WS2, *brs1-1* and *brs1-1D*. The height of the pink circled area represents the level of protein expression. **c** The protein quantification of spot 6 and 7 in WS2, *brs1-1* and *brs1-1D*. The Y-axis represents the normal volume of differential protein spots. All results are shown as mean ± standard deviation (SD) from three biological replicates. The asterisks indicate a statistically significant difference (Student’s t-test, *p < 0.05)

To further understand the function of BRS1, proteomic analysis was performed on WS2, *brs1-1* and *brs1-1D* seedlings using two-dimensional fluorescence difference gel electrophoresis (2D-DIGE) (Fig. 6a). Nineteen proteins were assigned as differentially expressed proteins (t-test, *p* value < 0.01), and 15 proteins were identified, of which 5 proteins were associated with redox regulation (Table 1), supporting that BRS1 is involved in redox regulation.

**Table 1:** List of differentially expressed proteins identified by mass spectrometry (MS)

No	Chromosome locus	Matched protein	Biological process	Expression Fold
1	AT3G08590	2,3-Biphosphoglycerate-independent phosphoglycerate mutase 2	Carbohydrate metabolism	1.18
2	AT3G57610	Adenylosuccinate synthetase	AMP biosynthesis	0.87
3	AT3G54050	Chloroplastic fructose 1,6-bisphosphate phosphatase	Fructose metabolism	0.88
4	AT2G39730	Rubisco activase	Light activation of rubisco	1.21
5	AT5G15650	UDP-arabinose mutase	Arabinose metabolism	0.68
6*	**AT1G65930**	**Cytosolic NADP + -dependent isocitrate dehydrogenase**	**Redox**	**0.22**
7*	**AT1G65930**	**Cytosolic NADP + -dependent isocitrate dehydrogenase**	**Redox**	**3.12**
**8**	**AT1G03475**	**Coproporphyrinogen III oxidase**	**Redox**	**1.15**
9	AT5G09530	Proline-rich protein 10	Seed germination	1.24
10	AT1G66200	Cytosolic glutamate synthetase	Glutamine biosynthesis	1.79
11	ATCG00490	Ribulose-bisphosphate carboxylase	Carbon fixation of photosynthesis	0.79
12	AT3G44310	Nitrilase 1	Nitrogen compound metabolism	1.37
**13**	**AT1G75280**	**Isoflavone reductase**	**Redox**	**1.26**
**14**	**AT1G78380**	**Glutathione s-transferase tau 19**	**Redox**	**1.16**
15	AT1G20340	DNA-damage resistance protein 112	Response to UV	0.67

The differentially expressed proteins were assigned according to the* p* < 0.01 (Student’s *t*-test, two tails). Proteins in bold refer proteins are involved in redox. ‘Expression Fold’ is calculated as the ratio of the expression level in *brs1-1D* to the control WS2. The asterisks indicate two differentially expressed proteins of cICDH. Triplicate biological repeats were performed with independent protein preparations

Notably, two protein spots (6 and 7) with significantly changed intensities compared with wild type (Fig. 6b), showing a decrease and increase in expression in *brs1-1D*, respectively (Fig. 6c), but corresponded to the same protein, the cytosolic NADP + -dependent isocitrate dehydrogenase (cICDH, at1g65930) (Table 1), a critical redox regulator [18]. Together, these data suggests that BRS1 plays important role in redox regulation.

### BRS1 participates in redox regulation by interacting with cICDH

**Fig. 7:**
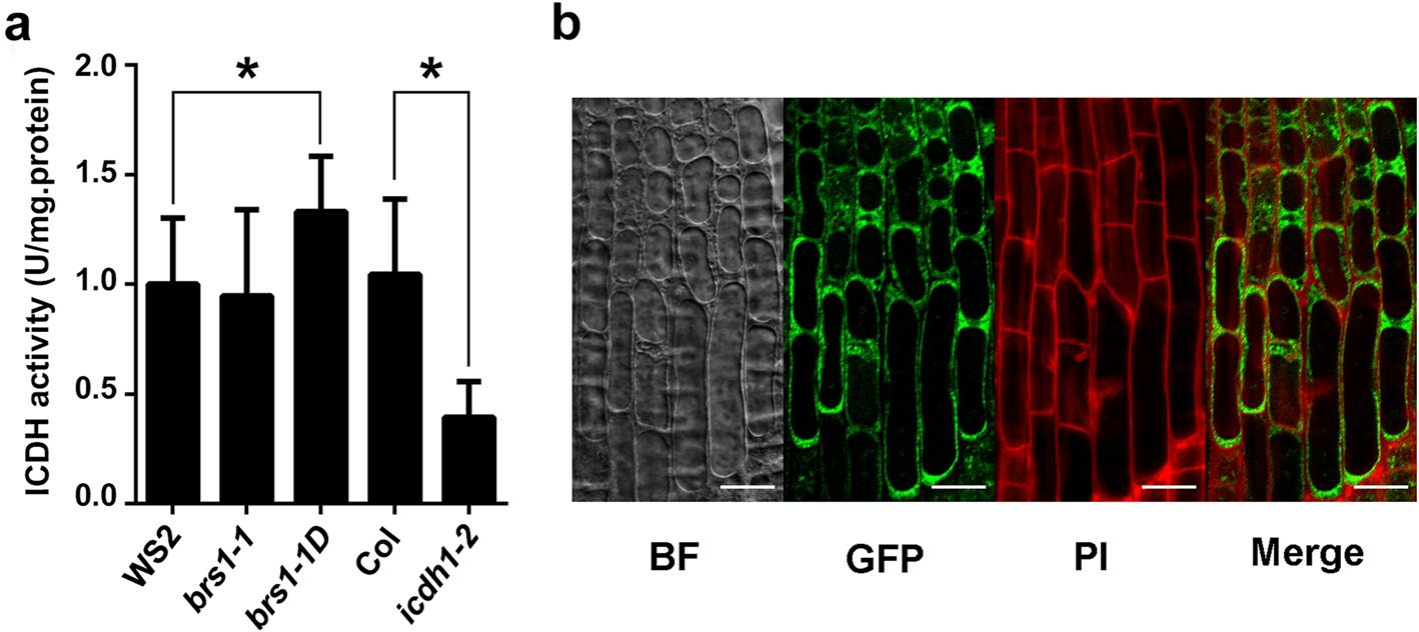
The enzyme activity of cICDH depends on BRS1. **a** Total extractable ICDH activity from wild type (WS2 and Col-0) seedlings and a range of mutant lines: *brs1-1D*, *brs1-1* and *icdh-2*. Means and SD of eight independent extracts are shown. Asterisks indicate a statistically significant difference based on Student’s *t*-test (*p < 0.05). **b** Subcellular localization of *BRS1*. Root tips from transgenic Col-0 plants expressing 35S-BRS1-GFP were used to visualize the subcellular localization of BRS1-GFP by confocal microscopy. The green fluorescence indicates the BRS1-GFP and the red fluorescence indicates propidium iodide staining. The merge channel represents the merge of BRS1-GFP and propidium iodide staining. Scale bar = 20 μm

To confirm that BRS1 regulates cICDH, the enzyme activity of cICDH was measured in *BRS1* mutants. We found that cICDH activity was significantly increased in *brs1-1D* compared to wild type plants (Fig. 7a), whilst no change was observed in *brs1-1* plants. These findings were consistent with our earlier results that only overexpression of *BRS1* can alter transcription and phenotypes. Altogether, this suggests that BRS1 regulate the activity of cICDH.

To investigate how the secretory protein BRS1 regulate cICDH, which is localized to the cytosol [19, 20], the cellular localization of BRS1 was evaluated. We observed the expression of BRS1 at the membrane and cell wall (Fig. 7b), which was consistent with the localization to the extracellular space [4, 5], indicating there is not a physical contact between BRS1 and cICDH. Consistently, yeast two-hybrid and pull-down assays found no direct interaction between cICDH and BRS1 (Table S2), indicating that BRS1 regulates cICDH indirectly.

## Discussion

BRS1, as a member of SCP, its role is relative clear in SCP family, and its function in plant growth and development has been demonstrated previously [5–7]. In this study, the transcriptomic and proteomic analyses demonstrate BRS1 also has a broad role in biotic and abiotic stress response.

Our results suggest that SA and JA signaling, which are key pathways required for the induction of plant defenses in response to pathogens and insects [21, 22], also contribute to the BRS1-related stress response (Fig. 3a). Similarly, an apoplastic SCPL in rice, OsBISCPL1, also induced stress responses via SA and JA signaling [9], indicating SCPLs may regulate stress responses in a same mechanism.

The trigger effector production by apoplastic proteases used be the key mechanism to induce plant stress response [13]. Our results shows that both cold sensitivity and cold tolerance are significantly enhanced with *BRS1* overexpression, then the underlying mechanism need be further investigated to clarify the precise role of BRS1 in cold perception and/or cold signaling.

Consistent with this notion, we found BRS1 participates in regulating redox homeostasis by indirectly interacting cICDH. The ICDH catalyze the production of NADPH, which is important for redox regulated cell metabolism and promoting redox signaling in response to oxidative stress [23, 24]. cICDH is responsible for more than 90% of total ICDH activity [23, 25]. Therefore, this enzyme plays a crucial role in maintaining redox homeostasis in the cell, and consequently, defense responses [18]. Exactly how BRS1 participates in this pathway still needs further investigation. Moreover, the two isoforms of ICDH showed similar molecular weights but different isoelectric points, implying they may be the different forms produced by protein modification, as described in previous report [26].

The knockout mutant of *BRS1* does not have any significant phenotypes [5–7], consistently, no DEGs were identified in the mutant *brs1-1* (Table S1). The existence of multiple redundant homologs in Arabidopsis may explain this result. In contrast, we observed many phenotypes [5–7] and identified significant alterations in gene expression upon overexpression of *BRS1* in this study. The novel splice variant identified in this study provides a new explanation for significant changes in phenotypes and transcriptional changes in *brs1-1D.* However, it still needs further investigations to clarify.

## Conclusions

In this study, transcriptomic and proteomic analyses revealed that BRS1 plays a role in regulating plant responses to cold stress and redox response. We found that BRS1 likely participates in redox regulation in cells through indirect interaction with cICDH. Altogether, our work sheds new light on the roles of SCPs in biotic and abiotic stress responses.

## Methods

### Plant Materials and Growth Condition

The plant materials used in this study are as follows: Wild-type Wassilewskija (WS2) and mutants of *BRS1* (*brs1-1* and *brs1-1D*). The *brs1-1D* is generated by crossing WS2 with an activation-tagging line *bri1-5 brs1-1D* (CS6127) from the Dr. Jia Li (Lanzhou University, Lanzhou, China) [4, 5, 7]. The *brs1-1* obtained from the Wisconsin Arabidopsis knockout pool and identified insertion a thymidine at 533 bp in first intron in this study. T-DNA insertion mutant *icdh-2* (SALK_056247) was ordered from the Salk Institute collection of Arabidopsis Biological Resource Center (ABRC) and verified by genotyping as previously described [18]. Theses seeds were grown in growth chamber at 22 °C under 16 h light/8 h dark conditions (light intensity 100 μmol·m^−2^·s^−1^, humidity 60%) for two weeks after germination to perform phenotypic observation. Seedlings of the three materials were cultured on half-strength Murashige and Skoog (1/2 MS) agar medium (supplemented with 1% (w/v) sucrose and 0.8% (w/v) agar, PH 5.6–5.8) in the same chamber and culture conditions, the total protein and RNA were extracted seven days after germination.

### RNA Extraction and Gene Expression Profiling

Seven-day-old WS2, *brs1-1*, and *brs1-1D* seedlings grown vertically on a 1/2 MS plate were frozen in liquid nitrogen, and triplicate of each material were collected. Total RNA was extracted from whole seedlings using a Tiangen RNAprep pure Plant Kit, and its quality was evaluated with Thermo Scientific NanoDrop2000. The RNA sequencing was completed by Biomarker company (Beijing, China).

### Identification of Differentially Expressed Genes (DEGs) and GO enrichment analysis

Raw transcriptome sequencing data cleaned using Trimmomatic (v 0.36). The clean reads were aligned to the *Arabidopsis thaliana* reference genome (TAIR 10) using HISAT2 (v 2.1.0) and the expression of genes were profiled using StringTie (v1.3.3) [27, 28].

Identification all of differential genes in *brs1-1* and *brs1-1D* compared to control WS2, respectively. The corrected read count data of genes were imported into the R package DESeq2 (v1.26.0) [29] to identify DEGs with the standard of a fold change ≥ 2.0, a false discovery rate (FDR)‐adjusted *p*‐value < 0.05, and expression (FPKM ≥ 1) in at least one sample for each comparison.

The GO descriptions were obtained by AnnotationHub (“AH75734”), and used the R package clusterProfiler (v3.14.0) [30] with the “enrichGO” function for GO enrichment analysis. The statistical significance of the enrichment of GO was examined using the hypergeometric distribution test, followed by multiple‐test correction using the Benjamini–Hochberg method. GO terms with q -value < 0.01 for further analysis.

### Alternative splicing analyses

Clean reads from all samples were aligned to the *Arabidopsis thaliana* reference genome (TAIR 10) using STAR (v 2.7.3a) [31]. Regtools (v0.5.2) were used to extract exon-exon junctions from RNA-seq BAM files with the parameters (-a 8 -m 50 -M 500,000). Then we used leafcutter (v 0.2.9) [32] to perform intron clustering (-m 50 -l 500,000) and differential splicing analyses (-i 3). Differential splicing of intron clusters was measured as the “change of percent spliced in” (∆PSI), The differential alternative splicing events were identified with |ΔPSI|> 0.05 and FDR-adjusted *P*-value < 0.01.

### Protein Preparation for Two-Dimensional Fluorescence Difference Gel Electrophoresis (2D-DIGE) Analysis

Seven-day-old seedlings (1 g) were harvested and ground into fine powder in liquid nitrogen and further mixed with 4 mL ice-cold extract buffer (20 mM Tris–HCl, PH 8.0, 1 mM EDTA, 20 mM NaCl, 5 mM MgCl_2_, 10 mM DTT, 2 mM phenylmethanesulfonyl fluoride, 1 μg/mL leupeptin, 10 μg/mL aprotinin, 1 μg/mL chymostatin and 1% phosphorylase inhibitor mixture). The supernatant was collected by centrifugation at 18,000 g for 20 min at 4 °C, and the pellet was resuspended in 3 mL extract buffer for repeat extraction. The combined supernatant was supplemented with chilled acetone to 80% (V/V) (4 times volume acetone of the supernatant) and incubated at -20 °C overnight to precipitate proteins. Proteins were pelleted by dissolved in 100 μL lysis buffer (7 M urea, 2 M thiourea, 4% w/v CHAPS, 20 mM Tris–HCl, pH 8.5), and the debris was removed by centrifugation at 18,000 g for 20 min. Finally, the pH of protein samples was adjusted to 8.5 with HCl and NaOH, and the concentration of proteins via Bio-Rad Bradford method using BSA as a standard [33]. The final proteins underwent 2D-DIGE immediately or were stored in aliquots at -80 °C. For each sample, at least quadruplicate protein preparations were performed.

### 2D-DIGE and Image Analysis

According to the manufacturer's instructions (GE Healthcare), the equivalent amounts of *brs1-1* and *brs1-1D* proteins were labeled with Cy3 and Cy5 minimum fluorescent dyes (400 pmol dye/50 μg protein), respectively. The internal standard WS2 protein was labeled with CY2 and mixed with two different labeled proteins in equal amounts. Adjust the mixed-labeled protein to a total volume of 450 μL with rehydration buffer (8 M urea, 13 mM DTT, 4% w / v CHAPS, 0.5% Pharmalyte pH 3–10), and then load on an IPG test strip holder containing an IPG test strip with 24 cm pH 4–7 linear gradient (GE Healthcare). Experimental methods of isoelectric focusing and SDS-PAGE as previously described [34]. To minimize systemic and inherent biological differences, it is recommended to combine four independent protein preparations for each sample [35].

Fluorescent images of gels were scanned by Typhoon 9400 scanner (GE Healthcare) and the images were analyzed using DeCyder 6.5 software in accordance with the DeCyder User Manual (GE Healthcare) [34]. Approximately 2000 spots were detected in each image, and then spots that showed significant differential expression were determined by ANOVA and Student's t-test (*p* < 0.05). 19 spots with significant differential expression were selected for mass spectrometric identification.

### Protein Identification

Coomassie brilliant blue staining was performed on the scanned 2-D-DIGE gel, and then differential protein spots were found by position comparison, but it was difficult to detect proteins with low background expression. Therefore, a 2-DE gel prepared with 1 mg of internal standard protein was used for staining to show spots that could not be determined from the 2D-DIGE gel.

After 19 differential protein spots were excised from 2-D-DIGE gel, each spot was destained in destaining buffer (25 mM ammonium bicarbonate, 50% v/v acetonitrile). Destained spots were dehydrated by acetonitrile and spun-dry, and digested with sequencing grade modified trypsin (Roche) at 37℃ for 16 h. The matrix-assisted laser-desorption ionization (MALDI) mass spectra were produced on an Ultroflex II MALDI time-of-flight/time-of-flight mass spectrometer (MALDI-TOF/TOF MS) (Bruker Daltonics, Germany) with use of FlexAnalysis 2.4 software. After tryptic peptide masses were transferred to a BioTools 3.0 interface (Bruker Daltonics), peptide mass fingerprintings (PMFs) were searched against the NCBInr protein database (http://www.ncbi.nlm.nih.gov/; NCBInr 20,071,214; 5,742,110 sequences) by use of Mascot software2.2.03(http://www.matrixscience.com; Matrix Science, London,U.K.).

### Enzyme activities

Seedlings (0.1 g) at 7 days after germination were ground into fine powder in liquid nitrogen and mixed with the extract buffer (1 mL 0.1 M NaH_2_PO_4_ (pH 8.0), 5 mM MgCl_2_, 14 mM 2-mercaptoethanol). Vortex the homogenate, centrifuge at 12,000 g for 5 min to remove insoluble materials, and measure ICDH activity by spectrophotometry [18, 20]. Determination of protein concentration as previously described Bio-Rad Bradford method using BSA as a standard [33].

### Confocal imaging

For protein localization of BRS1, the corresponding seedlings root tips from transgenic Col-0 plants expressing 35S-BRS1-GFP were stained in 0.1 mg/ml propidium iodide for 8 min. Seedlings were photographed using a confocal fluorescence microscope (Leica, TCS SP8). GFP was excited with a 488 nm laser and detected at 495–535 nm. Propidium iodide was excited with a 552 nm laser and detected at 530–680 nm. Images were further analyzed using Adobe Photoshop and ImageJ.

## Supplementary Information

**Additional file 1: Table S1.** Identification of DEGs in *BRS1* mutants. **Table S2. **Proteins identified by BRS1-GFP pull-down.

**Additional file 2: Figure S1. **The seedling hypocotyl phenotypes of WS2, *brs1-1* and *brs1-1D*. **a.** The seedling phenotypes of WS2, *brs1-1* and *brs1-1D* grown on 1/2 MS medium under long-day conditions. Photos captured 7 DAG. Scale bar = 1 cm. **b. **The hypocotyl phenotypes of WS2,* brs1-1* and *brs1-1D* seedlings grown on 1/2 MS medium in the dark. Photos captured 5 DAG. Scale bar = 1 cm. **c.** Comparison of hypocotyl lengths in **(a)**. Means ± SD are shown from three independent experiments, n≥ 20 in each experiment. The asterisks indicate a statistically significant difference (Student’s *t*-test, *p < 0.05). **d.** Comparison of the hypocotyl lengths in** (b)**. Means ± SD are shown from three independent experiments, n ≥ 20 in each experiment. The asterisks indicate a statistically significant difference (Student’s *t*-test, *p < 0.05). **Figure S2. **The expression analysis of *BRS1* homologs in wild type and BRS1 mutants. RNA sequencing was used to calculate the expression levels (FPKM value) of five *BRS1* homologs: *SCPL22*, *SCPL23*, *SCPL25*, *SCPL26* and *SCPL27* in WS2, *brs1-1* and *brs1-1D *plants. Mean ± SD is shown. The asterisks indicate a statistically significant difference (Student’s *t*-test, *p < 0.05).

## Data Availability

All data generated or analyzed in this study are included in this article and the supplemental files. The raw data of RNA sequencing were submitted to the NCBI database with the bioproject ID: PRJNA657702.

## Abbreviations

SCP: Serine carboxypeptidase
BRS1: Brassinosteroid-insensitive 1 suppressor 1
ROS: Reactive oxygen species
DEGs: Differentially expressed genes
FPKM: Fragments per kilobase of exon per million fragments mapped
SA: Salicylic acid
JA: Jasmonic acid
cICDH: Cytosolic NADP + -dependent isocitrate dehydrogenase

## References

[1] Fraser CM, Rider LW, Chapple C (2005). An expression and bioinformatics analysis of the arabidopsis serine carboxypeptidase-like gene family. Plant Physiol.

[2] Tripathi LP, Sowdhamini R (2006). Cross genome comparisons of serine proteases in arabidopsis and rice. BMC Genomics.

[3] Zhu D, Chu W, Wang Y, Yan H, Chen Z, Xiang Y (2018). Genome-wide identification, classification and expression analysis of the serine carboxypeptidase-like protein family in poplar. Physiol Plant.

[4] Zhou A, Li J (2005). Arabidopsis BRS1 is a secreted and active serine carboxypeptidase. J Biol Chem.

[5] Li J, Lease KA, Tax FE, Walker JC (2001). BRS1, a serine carboxypeptidase, regulates BRI1 signaling in *Arabidopsis thaliana*. Proc Natl Acad Sci U S A.

[6] Wen J, Li J, Walker J (2012). Overexpression of a serine carboxypeptidase increases carpel number and seed production in *Arabidopsis thaliana*. Food and Energy Security.

[7] Deng Q, Wang X, Zhang D, Wang X, Feng C, Xu S (2017). BRS1 function in facilitating lateral root emergence in Arabidopsis. Int J Mol Sci.

[8] Moura DS, Bergey DR, Ryan CA (2001). Characterization and localization of a wound-inducible type I serine-carboxypeptidase from leaves of tomato plants (*Lycopersicon esculentum* Mill.). Planta.

[9] Liu H, Wang X, Zhang H, Yang Y, Ge X, Song F (2008). A rice serine carboxypeptidase-like gene *OsBISCPL1* is involved in regulation of defense responses against biotic and oxidative stress. Gene.

[10] Domínguez F, González MC, Cejudo FJ (2002). A germination-related gene encoding a serine carboxypeptidase is expressed during the differentiation of the vascular tissue in wheat grains and seedlings. Planta.

[11] Zhu JK (2016). Abiotic stress signaling and responses in plants. Cell.

[12] Zhou JM, Zhang Y (2020). Plant Immunity: danger perception and signaling. Cell.

[13] Wang Y, Wang Y, Wang Y (2020). Apoplastic proteases - powerful weapons against pathogen infection in plants. Plant Commun.

[14] Chinnusamy V, Zhu J, Zhu JK (2007). Cold stress regulation of gene expression in plants. Trends Plant Sci.

[15] Msanne J, Lin J, Stone JM, Awada T (2011). Characterization of abiotic stress-responsive *Arabidopsis thaliana* RD29A and RD29B genes and evaluation of transgenes. Planta.

[16] Breton G, Danyluk J, Charron JB, Sarhan F (2003). Expression profiling and bioinformatic analyses of a novel stress-regulated multispanning transmembrane protein family from cereals and Arabidopsis. Plant Physiol.

[17] Kurkela S, Borg-Franck M (1992). Structure and expression of kin2, one of two cold- and ABA-induced genes of *Arabidopsis thaliana*. Plant Mol Biol.

[18] Mhamdi A, Mauve C, Gouia H, Saindrenan P, Hodges M, Noctor G (2010). Cytosolic NADP-dependent isocitrate dehydrogenase contributes to redox homeostasis and the regulation of pathogen responses in Arabidopsis leaves. Plant Cell Environ.

[19] Gálvez S, Hodges M, Decottignies P, Bismuth E, Lancien M, Sangwan RS, Dubois F, LeMaréchal P, Crétin C, Gadal P (1996). Identification of a tobacco cDNA encoding a cytosolic NADP-isocitrate dehydrogenase. Plant Mol Biol.

[20] Galvez S, Bismuth E, Sarda C, Gadal P (1994). Purification and characterization of chloroplastic NADP-Isocitrate dehydrogenase from mixotrophic tobacco cells (comparison with the cytosolic isoenzyme). Plant Physiol.

[21] Browse J (2009). Jasmonate passes muster: a receptor and targets for the defense hormone. Annu Rev Plant Biol.

[22] Zhang Y, Li X (2019). Salicylic acid: biosynthesis, perception, and contributions to plant immunity. Curr Opin Plant Biol.

[23] Marino D, González EM, Frendo P, Puppo A, Arrese-Igor C (2007). NADPH recycling systems in oxidative stressed pea nodules: a key role for the NADP+ -dependent isocitrate dehydrogenase. Planta.

[24] Hodges M, Flesch V, G S, Bismuth E. Higher plant NADP+-dependent isocitrate dehydrogenases, ammonium assimilation and NADPH production. Plant Physiol Biochem 2003; 41(6–7):577–85.

[25] Hodges M (2002). Enzyme redundancy and the importance of 2-oxoglutarate in plant ammonium assimilation. J Exp Bot.

[26] Tang W, Kim TW, Oses-Prieto JA, Sun Y, Deng Z, Zhu S, Wang R, Burlingame AL, Wang ZY (2008). BSKs mediate signal transduction from the receptor kinase BRI1 in Arabidopsis. Science.

[27] Kim D, Langmead B, Salzberg SL (2015). HISAT: a fast spliced aligner with low memory requirements. Nat Methods.

[28] Pertea M, Pertea GM, Antonescu CM, Chang TC, Mendell JT, Salzberg SL (2015). StringTie enables improved reconstruction of a transcriptome from RNA-seq reads. Nat Biotechnol.

[29] Love MI, Huber W, Anders S (2014). Moderated estimation of fold change and dispersion for RNA-seq data with DESeq2. Genome Biol.

[30] Yu G, Wang L, Han Y, He Q (2012). ClusterProfiler: an R package for comparing biological themes among gene clusters. OMICS.

[31] Dobin A, Davis CA, Schlesinger F, Drenkow J, Zaleski C, Jha S, Batut P, Chaisson M, Gingeras TR (2013). STAR: ultrafast universal RNA-seq aligner. Bioinformatics.

[32] Li YI, Knowles DA, Humphrey J, Barbeira AN, Dickinson SP, Im HK, Pritchard JK (2018). Annotation-free quantification of RNA splicing using leafcutter. Nat Genet.

[33] Bradford MM (1976). A rapid and sensitive method for the quantitation of microgram quantities of protein utilizing the principle of protein-dye binding. Anal Biochem.

[34] Xu SB, Yu HT, Yan LF, Wang T (2010). Integrated proteomic and cytological study of rice endosperms at the storage phase. J Proteome Res.

[35] Barceló-Batllori S, Kalko SG, Esteban Y, Moreno S, Carmona MC, Gomis R (2008). Integration of DIGE and bioinformatics analyses reveals a role of the antiobesity agent tungstate in redox and energy homeostasis pathways in brown adipose tissue. Mol Cell Proteomics.

