# Supplementary figures and images for: BRS1 mediates plant redox regulation and cold responses
Source: BMC Plant Biol. 2021 Jun 11;21:268. doi: 10.1186/s12870-021-03045-y (PMC8193866; doi:10.1186/s12870-021-03045-y)

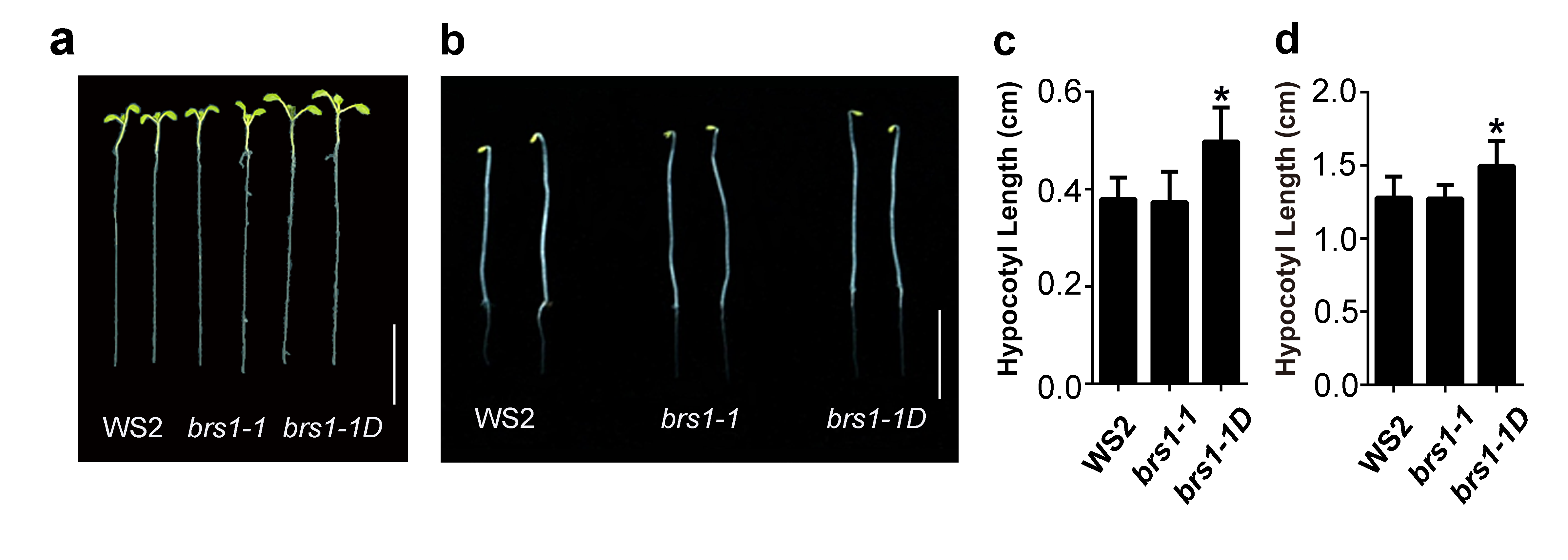

Supplement: Supplementary file 2 — Additional file 2: Figure S1. The seedling hypocotyl phenotypes of WS2, brs1-1 and brs1-1D. a. The seedling phenotypes of WS2, brs1-1 and brs1-1D grown on 1/2 MS medium under long-day conditions. Photos captured 7 DAG. Scale bar = 1 cm. b. The hypocotyl phenotypes of WS2, brs1-1 and brs1-1D seedlings grown on 1/2 MS medium in the dark. Photos captured 5 DAG. Scale bar = 1 cm. c. Comparison of hypocotyl lengths in (a). Means ± SD are shown from three independent experiments, n≥ 20 in each experiment. The asterisks indicate a statistically significant difference (Student’s t-test, *p < 0.05). d. Comparison of the hypocotyl lengths in (b). Means ± SD are shown from three independent experiments, n ≥ 20 in each experiment. The asterisks indicate a statistically significant difference (Student’s t-test, *p < 0.05). Figure S2. The expression analysis of BRS1 homologs in wild type and BRS1 mutants. RNA sequencing was used to calculate the expression levels (FPKM value) of five BRS1 homologs: SCPL22, SCPL23, SCPL25, SCPL26 and SCPL27 in WS2, brs1-1 and brs1-1D plants. Mean ± SD is shown. The asterisks indicate a statistically significant difference (Student’s t-test, *p < 0.05). [file 12870_2021_3045_MOESM2_ESM.zip › Figure S1.tif]

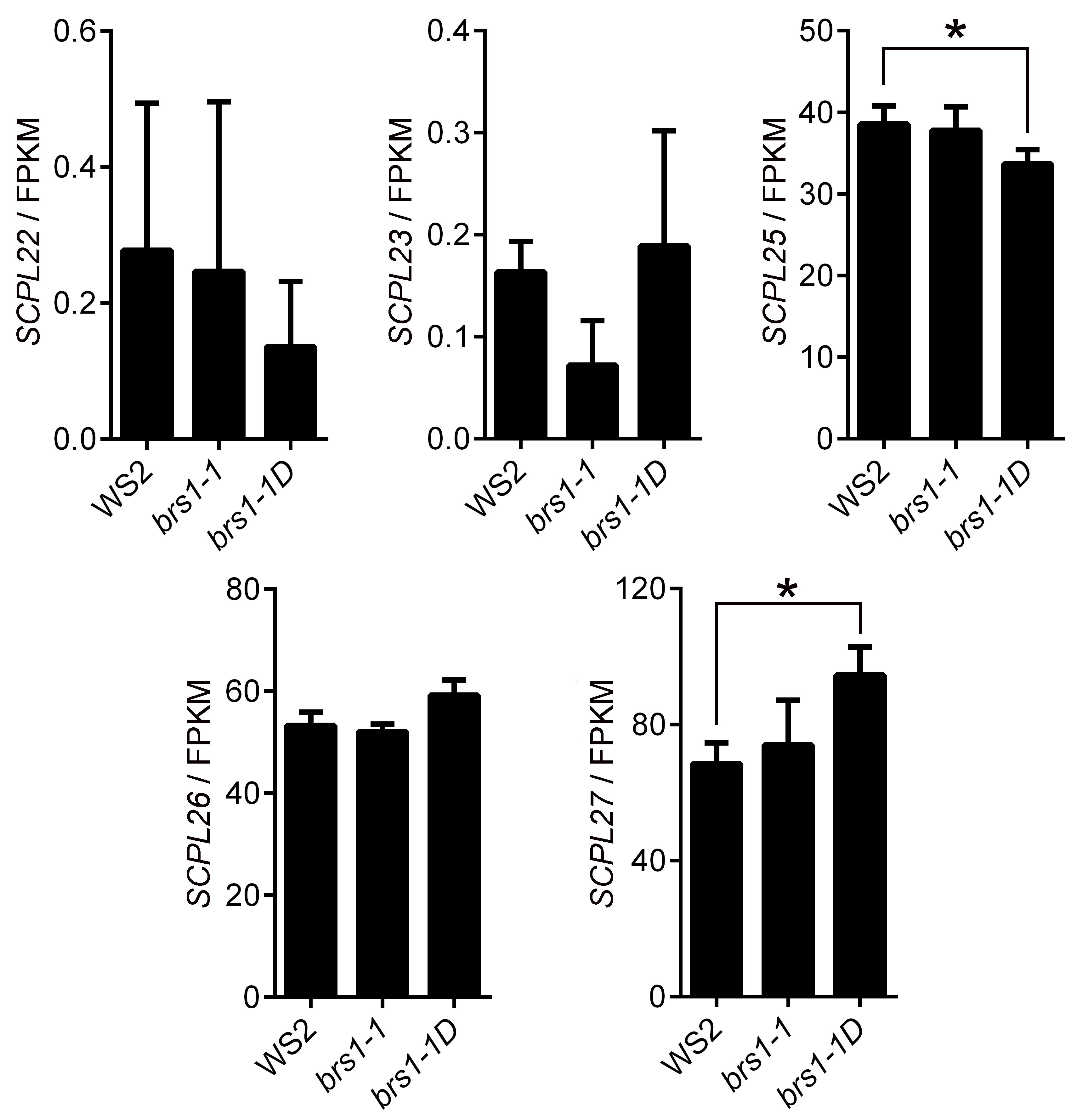

Supplement: Supplementary file 2 — Additional file 2: Figure S1. The seedling hypocotyl phenotypes of WS2, brs1-1 and brs1-1D. a. The seedling phenotypes of WS2, brs1-1 and brs1-1D grown on 1/2 MS medium under long-day conditions. Photos captured 7 DAG. Scale bar = 1 cm. b. The hypocotyl phenotypes of WS2, brs1-1 and brs1-1D seedlings grown on 1/2 MS medium in the dark. Photos captured 5 DAG. Scale bar = 1 cm. c. Comparison of hypocotyl lengths in (a). Means ± SD are shown from three independent experiments, n≥ 20 in each experiment. The asterisks indicate a statistically significant difference (Student’s t-test, *p < 0.05). d. Comparison of the hypocotyl lengths in (b). Means ± SD are shown from three independent experiments, n ≥ 20 in each experiment. The asterisks indicate a statistically significant difference (Student’s t-test, *p < 0.05). Figure S2. The expression analysis of BRS1 homologs in wild type and BRS1 mutants. RNA sequencing was used to calculate the expression levels (FPKM value) of five BRS1 homologs: SCPL22, SCPL23, SCPL25, SCPL26 and SCPL27 in WS2, brs1-1 and brs1-1D plants. Mean ± SD is shown. The asterisks indicate a statistically significant difference (Student’s t-test, *p < 0.05). [file 12870_2021_3045_MOESM2_ESM.zip › Figure S2.tif]
